# 

**Published:** 1882-11

**Authors:** 


### TABLE OF CONTENTS FOR NOVEMBER, 1882.

### ORIGINAL COMMUNICATIONS.

PAGE.
ART. I.—A Winter Cold. *By* F. H. BOSWORTH, M. D. .......... 593

### SELECTIONS.

Male Sexual Hemicrania .......... 601
A Case of Hydatids of the Lungs .......... 603
Dysmenorrhea .......... 605
The Antiseptic Treatment of Typhoid Fever .......... 606
Sick Headache .......... 607
The Positive Treatment of Gonorrhea .......... 608
Damiana—Unexpected Results from its Use in a Case .......... 610
Studies on Myxo-Angioma of the Skin .......... 612
Typhoid Fever Epidemic in Paris .......... 614
Catarrhal Ozœna .......... 614
Anatomical Basis of Tetanus .......... 614
Successful Midwifery—Baby Incubator .......... 615
Hot Pack in Puerperal Eclampsia .......... 615
Nitrate of Silver to Prevent Ophthalmia Neonatorum .......... 615
EDITORIAL .......... 616
CURRENT NEWS AND OPINION .......... 616
POPULAR SCIENCE DEPARTMENT .......... 617

